# Multiwave pandemic dynamics explained: how to tame the next wave of infectious diseases

**DOI:** 10.1038/s41598-021-85875-2

**Published:** 2021-03-23

**Authors:** Giacomo Cacciapaglia, Corentin Cot, Francesco Sannino

**Affiliations:** 1 Institut de Physique des 2 Infinis (IP2I), CNRS/IN2P3, UMR5822, 69622 Villeurbanne, France; 2 Université de Lyon, Université Claude Bernard Lyon 1, 69001 Lyon, France; 3 CP3-Origins & The Danish Institute for Advanced Study, University of Southern Denmark, Campusvej 55, 5230 Odense, Denmark; 4 Dipartimento di Fisica E. Pancini, Università di Napoli Federico II & INFN sezione di Napoli, Complesso Universitario di Monte S. Angelo Edificio 6, via Cintia, 80126 Naples, Italy

**Keywords:** Scientific data, Health policy

## Abstract

Pandemics, like the 1918 Spanish Influenza and COVID-19, spread through regions of the World in subsequent waves. Here we propose a consistent picture of the wave pattern based on the epidemic Renormalisation Group (eRG) framework, which is guided by the global symmetries of the system under time rescaling. We show that the rate of spreading of the disease can be interpreted as a time-dilation symmetry, while the final stage of an epidemic episode corresponds to reaching a time scale-invariant state. We find that the endemic period between two waves is a sign of instability in the system, associated to near-breaking of the time scale-invariance. This phenomenon can be described in terms of an eRG model featuring complex fixed points. Our results demonstrate that the key to control the arrival of the next wave of a pandemic is in the strolling period in between waves, i.e. when the number of infections grows linearly. Thus, limiting the virus diffusion in this period is the most effective way to prevent or delay the arrival of the next wave. In this work we establish a new guiding principle for the formulation of mid-term governmental strategies to curb pandemics and avoid recurrent waves of infections, deleterious in terms of human life loss and economic damage.

Pandemics, like the 1918 Spanish Influenza^1^ and COVID-19, spread through regions of the World in subsequent waves. There is, however, no consensus on the origin of this pattern, which may originate from human behaviour rather than from the virus diffusion itself. Time-honoured models of the SIR type^2^ or others based on complex networks^3–5^ describe well the exponential spread of the disease, but cannot naturally accommodate the wave pattern. Nevertheless, understanding this time-structure is of paramount importance in designing effective prevention measures. Here we propose a consistent picture of the wave pattern based on the epidemic Renormalisation Group (eRG) framework^6,7^, which is guided by the global symmetries of the system under time rescaling. We show that the rate of spreading of the disease can be interpreted as a time-dilation symmetry, while the final stage of an epidemic episode corresponds to reaching a time scale-invariant state. We find that the endemic period between two waves is a sign of instability in the system, associated to near-breaking of the time scale-invariance. This phenomenon can be described in terms of an eRG model featuring complex fixed points^8^. Our results demonstrate that the key to control the arrival of the next wave of a pandemic is in the strolling period in between waves, i.e. when the number of infections grows linearly. Thus, limiting the virus diffusion in this period is the most effective way to prevent or delay the arrival of the next wave. In this work we establish a new guiding principle for the formulation of mid-term governmental strategies to curb pandemics and avoid recurrent waves of infections, deleterious in terms of human life loss and economic damage.

As it emerged from the Spanish Influenza that hit the World in three consecutive waves between spring 1918 and the early months of 1919, virus-driven pandemics can feature a wave pattern, even though the origin of this behaviour is not understood^1^. The very recent pandemic, caused by the coronavirus SARS-CoV-2, is showing a similar pattern, with a first wave hitting in the spring of 2020, and following ones still raging various regions of the World. Reliable algorithms were used at the beginning of the pandemic to predict the evolution of the number of cases affected by the COVID-19 disease^9–11^, however it has proven difficult to predict the arrival of a second wave in the fall 2020^12^. With the exception of a few countries like China, Vietnam and New Zealand, all regions of the World are suffering from multiple waves of COVID-19 infections.

The diffusion of the virus can be described by various time-honoured models, like compartmental models of the SIR type^2^ and complex networks^3–5^. These mathematical frameworks account for the exponential growth of the number of new infected cases, and the slowing down of the spreading once most of the susceptible individuals are infected. However, it is not a simple task to generate a wave pattern. For instance, in SIR models, one could induce a second wave either by injecting by hand new individuals in the susceptible sub-population, or by including a probability that the removed individuals may return to the state of susceptible. The latter case cannot apply to the COVID-19, as very few cases of recovered individuals being infected again have been recorded.

In an article first posted at the beginning of August^13^, we successfully predicted the occurrence of a second wave in Europe starting in September–October. The analysis is based on the eRG framework^6^, extended by interactions between various countries^7^. The approach is based on the analysis of the time evolution of the total number of infected cases and the symmetries that this epidemic curve reveals, allowing to extract reliable information from the data independently on the specific conditions met in each country. In fact, all the elements that can influence the velocity of the disease spreading are included in a single parameter, which contains the effect of local conditions, non-pharmaceutical interventions and socio-demographical characteristics. The eRG, therefore, can provide complementary information to studies that analyse in detail the effect of various measures^14–18^. As an example, the eRG framework has been used to study the effect of mobility reduction in Europe and the US during the first wave^19^, highlighting a universal time-frame of 2–4 weeks before an observable effect can be detected in the virus diffusion. For comparison, detailed studies of the mobility in the US^20^ have been able to identify the locations and events that foster the infection of new individuals and ignite hotspots.

In this work we focus on the total number of infected cases, as this is the most reliable tracker of the time-evolution of the pandemics. In fact, other data, like the number of deaths and of hospitalisations, depend on factors like the age distribution and medical pre-conditions of the infected individuals, which can influence the delay between the infection and the time-stamp in the data. The master multiwave equation for the time-evolution of the total number of infected cases $I_{j} (t)$ in a region *j* reads:

1 $$\begin{aligned} - \beta _\text{multiwaves} (I_j) = \frac{1}{A_j} \frac{d I_j(t)}{\gamma _j\ d t} = \frac{I_j}{A_j} \left[ \left( 1 - \frac{I_j}{A_j} \right) ^2 - \delta _{j,0} \right] ^{p_{j,0}} \; \prod _{\rho =1}^w \left[ \left( 1-\zeta _{j,\rho } \frac{I_j}{A_j} \right) ^2 - \delta _{j,\rho } \right] ^{p_{j,\rho }} +\ \sum _l \frac{k_{jl}}{\gamma _j\ n_{m j}} \frac{ {I}_l - {I}_j}{A_j}, \end{aligned}$$

where the first term on the right-hand side is a generalisation for $w+1$ consecutive waves of the CeRG equations^8^ and the second term contains the interactions between regions^7^. Here, we will always consider the number of cases per million inhabitants in order to compare different regions. In the master equation, most of the parameters are explicitly independent on the normalisation, as $I_j(t)$ always appears divided by the total number of cases at the end of the first wave, $A_j$: the only exception is the interaction term, which also depends on the population of the regions ($n_{m j}$ measures the population of region-*j* in millions). The parameters $\gamma _j$ measure the effective velocity of the virus in each regions, and can be associated to an effective infection rate. This parameter can be eliminated from the equation by measuring the time in terms of a region-dependent scale, $\tau _j = \gamma _j t$, once the couplings $k_{jl}$ are also rescaled: $\gamma _j$ can therefore be interpreted as a local *time-dilation*, characteristic of each region and taking into account all the non-pharmaceutical measures and local conditions in each region. These parameters can be extracted from the data at the beginning of the epidemic diffusion in each region, independently on the normalisation of the number of cases, which is very sensitive to the testing strategies^21^ changing during the pandemic. More details on the equations, and on the meaning of other parameters can be found in the Supplementary Information.

The master equation (1) encodes the multiwave pattern in two ways: in the first term, the parameters $\delta _{j,\rho }$ destabilise the fixed points at $I_j (\tau ^*_\rho ) = A_j/\zeta _\rho$; in the second term, the interactions with other regions, or with an external source, can also destabilise the system and drive a new growth of $I_j$ towards the next fixed point. In fact, for $\delta _{j,\rho } = 0$, the number of infected will grow until $I_j (\tau _\rho ^*) = A_j/\zeta _\rho$, where the growth stops because of the vanishing of the beta function. This is a steady-state, independent of time, which signals the end of the infection. For $\delta _{j,\rho } < 0$, the zero is moved on the complex plane and cannot be reached, thus driving an endemic state with linear growth, which we call *strolling* in honour to the application of this formalism in high energy physics^8^. The second term has been used^13^ to predict the European second wave of September 2020, where $k_{jl}$ was associated to an estimated number of travellers between each country. In general, both effects are expected to be present: as we will see, the instability due to $\delta$ provides a maximal delay for the arrival of the next wave, which is directly related to the number of new cases recorded during the strolling period between waves. The presence of a large interaction can induce an early arrival of the new wave.

Thus, effective measures to prevent and control the next wave of a pandemic like COVID-19 can only go via a strict control of the number of cases inside the country or region, combined to effective tracking of new infected individuals traveling in. This is precisely the strategy followed by China, Vietnam and New Zealand, leading to an early extinction of the disease and the absence of a second wave.

## Results

**Figure 1:**
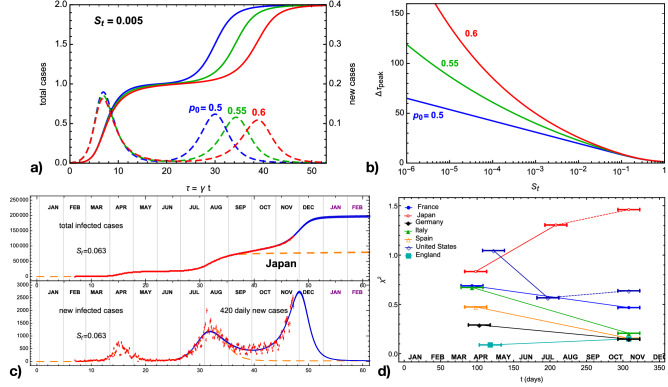
Illustration and validation of the CeRG multiwave model. The panel (**a**) shows sample solutions of the CeRG multiwave equation for $w=1$, with $\delta _1 = 0$, so that the epidemic episode is extinguished after two waves. The total number of cases, normalised to the first peak, is shown in solid, while the normalised new cases are shown in dashed. In the panel (**b**) we show the dependence of the delay between the two peaks of new infections, $\Delta \tau _\text{peak}$ measured in the local time, as a function of $S_\text{t}$. The CeRG parameters are fixed to the following values, unless specified: $p_0 = 0.5,\ p_1 = 0.65,\ \zeta _1 = 0.5$. Panel (**c**) shows the CeRG model applied to the second and third wave in Japan (blue) as compared to the data (red) and the eRG fits of the two waves (orange). In panel (**d**) we show the value of the geographical uniformity indicator as defined in the text for a sample of countries, showing that the virus is more equally spread in the various regions during the second wave, in most cases.

The CeRG multiwave model, corresponding to (1) without the interaction terms, can be used to effectively describe a pandemic episode in multiple subsequent waves. To illustrate the model, in the top panels of Fig. 1 we show some features of the solutions for an isolated region with only two waves, corresponding to $w=1$ and $\delta _1 = 0$. The latter condition ensures that the epidemic is extinguished after the second wave. All results are shown for number of cases normalised to the first wave, and expressed in the local time $\tau = \gamma t$. Panel (a) shows three solutions (solid lines) together with the corresponding new cases (dashed lines), for three values of $p_0$. We can clearly see the two-wave structure emerging in the solution, the fact that the second peak tends to be flatter than the first (for $\zeta _1 = 1/2$). Furthermore, larger values of the exponent $p_0$ tend to delay the second peak and flatten it. An important factor in controlling the arrival of a future wave is the number of new cases during the intermediate strolling phase, which we encode in the parameter

2 $$\begin{aligned} S_\text{t} = \left. \frac{1}{A \gamma } \frac{d\ I}{d\ t} \right| _\text{strol}, \end{aligned}$$

normalised to the total number of infected cases after the first wave, *A*, and expressed in terms of the local time. For the CeRG multiwave model in Eq. (1), the parameter $S_t$ for each interwave period can be expressed as

3 $$\begin{aligned} S_{t,k} = (-\delta _k)^{p_k} \prod _{\rho \ne k} (1-\zeta _\rho )^{p_\rho }, \end{aligned}$$

for the strolling phase after the *k*th wave. This is a crucial parameter in controlling the timing of the second wave, as illustrated in panel (b) of Fig. 1 where we show the peak delay $\Delta \tau _\text{peak}$ (in local time) as a function of $S_\text{t}$. For $S_\text{t} \rightarrow 1$, the delay goes to zero as the two waves merge into a single one, while it grows for smaller values following a power law that depends on $p_0$. The peak delays also depend strongly on $\zeta _1$, which encodes the height of the second peak, being more delayed for decreasing $\zeta _1$: for values above 0.8, however, the second wave becomes too small, thus the solution looses physical relevance.

**Figure 2:**
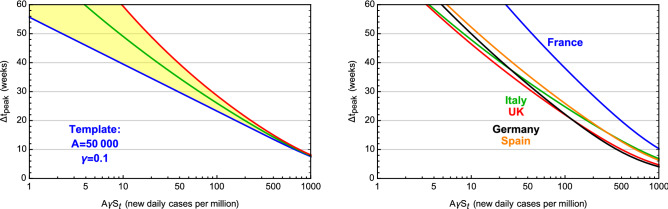
Strolling control to delay the next wave. Delay of the next peak (in real time weeks) as a function of the strolling severity, expressed in terms of the daily number of new infected cases per million inhabitants ($A \gamma S_t$). In the right panel, we show the results for the CeRG model applied to France, Italy, the UK, Germany and Spain. In the left panel we show the result for a template country with $A=50,000$ and $\gamma = 0.1$. The band is given by varying $p_0$ between 0.5 and 0.6.

To connect the result in Fig. 1b to a specific country or region, it is enough to fit the values of *A* and $\gamma$ from the current wave, and appropriately rescaling the values of $S_t$ and $\Delta \tau _\text{peak}$: the delay in real time is $\Delta t_\text{peak} = \Delta \tau _\text{peak}/\gamma$ while the new daily number of cases during the strolling is $A \gamma S_t$. In the left panel of Fig. 2 we provide a template for a country/region with $A=50,000$ and $\gamma = 0.1$ (inverse days). The band, which can be considered an error on the prediction, comes from varying $p_0$. This plot shows that controlling the number of daily new cases during the endemic strolling phase below 10 could be enough to push the next wave peak beyond 40 weeks. In the right panel we show the same plot for 5 European countries, obtained by fitting the epidemiological data of the current wave.

Comparing the predictions of the CeRG multiwave model to data is not an easy task: in fact, the number of detected infections, collected via the positivity of the tests done in each country, depends crucially on the number of tests done each day^21^ and on the specific testing policy adopted over time. For instance, during the first wave, many countries did fewer tests while focusing on hospitalised cases, while a more extensive testing campaign occurred starting in the summer months. As a consequence, quantities that depend on the number of cases, like the delay between peaks, cannot be computed accurately as a bias between two waves is present in the data. We, therefore, will apply the model to the second wave and following ones. As an example, in panel (c) of Fig. 1 we show the data for Japan compared to a scenario based on the CeRG multiwave model. Japan is an ideal candidate for this model since, being an island, the frontiers can be well-controlled, and one can consider the country as an isolated system. Furthermore, the second wave has already ended, followed by a two-month period of strolling with around 450 new infected cases detected each day. The CeRG model, shown in blue, provides a good quantitative and qualitative description of the data, and predicts that the third wave will peak at the beginning of December and be slightly higher than the second ($\zeta _1 = 0.4$). Between the first and second waves, instead, no significant strolling was observed. This scenario could be interpreted in the following way: after the first wave, the virus diffusion was strongly limited by the enacted measures. However, new infected cases may have entered the country from abroad and/or spontaneous emergence of local hotspots inside the country (parametrised by the *k*-interactions). After the second wave, the virus kept spreading geographically within the population triggering at a later stage the third wave. The latter phase can be described by the CeRG model, while the transition between the first and second wave is due to *external* interactions. We do not attempt to unify the three waves because of the bias in the counting.

Testing the geographical diffusion of the virus in each country can provide useful indications on the mechanism behind each wave. For this purpose, we define a uniformity indicator, $\chi ^2$, which encodes how far is the distribution of new cases in regions of the country from a completely flat one. Smaller values of $\chi ^2$ indicate a more uniform distribution. For Japan, we considered the new cases in the various prefectures (where we exclude Okinawa for the geographical distance from the mainland) during the first and second waves, as shown in red in Fig.1d. The second wave has a larger $\chi ^2$, which could be interpreted as more localised diffusion due to hotspots or travellers returning to their home cities. We also report the indicator for the month of November, which we would expect to be reduced if the strolling plays an important role in creating the third wave. The result is too preliminary, as Japan is still far from the peak of the third wave and the indicator is found to be minimised at the peak.

Having validated the CeRG approach, we can now apply it to understand and predict the next wave in various regions of the World. As a caveat, we should recall that the interactions between regions and the presence of hotspots can also affect the results and anticipate the insurgence of the next wave.

**Table 1:** Numerical results for the eRG fit of the current wave and the CeRG forecast for the next wave. Columns 2 and 3 contain the results for the fit of the current wave using the eRG model^6^, where *A* is given in number of cases per million inhabitants and $\gamma _\text{eRG}$ in inverse days. The current wave corresponds to the second one for all countries, except for South Africa, Bolivia and Saudi Arabia for which it is the first. In the last three columns we show the expected peak dates (within a 1 week error) of the current and next waves and the expected value of the number of daily new cases per million inhabitants $I'_\text{strol}$ during the inter-wave strolling period. Except for Australia, South Africa, Bolivia, Saudi Arabia, Japan and South Korea, we fix $S_\text{t} = 0.01$, $p_0 = 0.55$, $p_1 = 0.6$ and $\zeta _1 = 1/2$, while $\gamma$ and $A_0$ are chosen to fit the second wave (first for South Africa, Bolivia, Saudi Arabia). For countries currently in the strolling period, all the parameters of the CeRG model are fitted.

Country	eRG fits		CeRG forecast		
	Current wave		Current peak		Next peak
	*A*	$\gamma _\text{eRG}$	Date ($\pm 1$ w)	$I'_\text{strol}$	Date ($\pm 1$ w)
France	$61(6) \times 10^3$	0.048(5)	2020-11-07	35	2021-11-20
Italy	$41(2) \times 10^3$	0.075(8)	2020-11-17	37	2021-07-17
UK	$21.3(2) \times 10^3$	0.0717(8)	2020-11-08	17	2021-07-24
Germany	$20(3) \times 10^3$	0.058(2)	2020-12-13	14	2021-10-05
Spain	$30(4) \times 10^3$	0.067(1)	2020-10-31	22	2021-08-04
Switzerland	$40(4) \times 10^3$	0.10(1)	2020-11-04	70	2021-04-20
Netherlands	$26.1(2) \times 10^3$	0.0797(8)	2020-10-24	24	2021-06-19
Belgium	$40.7(4) \times 10^3$	0.121(2)	2020-10-24	70	2021-03-13
Denmark	$12(2) \times 10^3$	0.062(2)	2020-11-07	9	2021-09-11
Iceland	$9.9(1) \times 10^3$	0.087(2)	2020-10-10	12	2021-04-20
Canada	$16(2) \times 10^3$	0.046(5)	2020-12-07	71	2022-01-08
South Africa	11039(35)	0.0704(5)	2020-07-11	18	2021-01-06
Bolivia	12240(20)	0.0442(2)	2020-07-25	3.7	2021-07-28
Saudi Arabia	9560(20)	0.0447(3)	2020-06-20	9.2	2021-04-17
Australia	735(3)	0.1015(7)	2020-07-25	1	2021-04-17
Japan	434(5)	0.094(1)	2020-08-08	3.4	2020-12-02
South Korea	157(3)	0.136(6)	2020-08-26	0.7	2020-11-25

**Figure 3:**
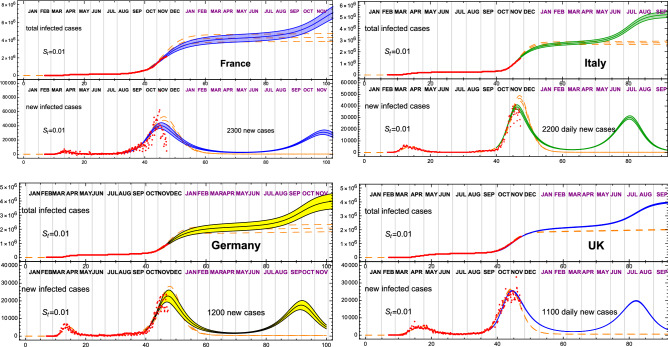
Strolling as a precursor of a COVID-19 third wave. Most European countries are still undergoing a second wave of COVID-19. In this figure we show how a strolling period, consistent on a fixed number of new infections per day (indicated in each panel) could lead to a third wave, as indicated by the solid band. The prediction corresponds to $S_\text{t} = 0.01$, and is compared to the data (adjourned to November 23) and the eRG fit from Table 1 (dashed orange). The plot includes France, Italy, Germany and the UK.

**Figure 4:**
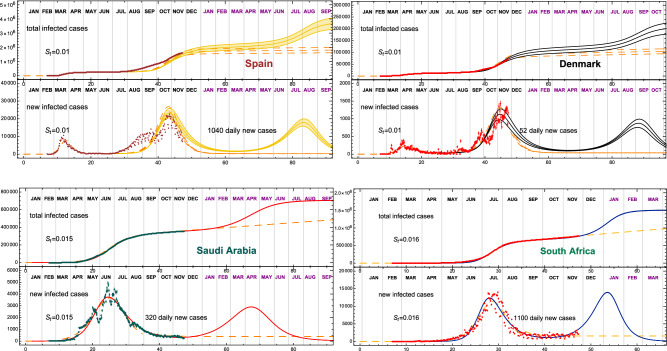
Strolling as a precursor of a new COVID-19 wave. The top panels shows Spain and Denmark, while the remaining European countries included in this study are shown in the Supplementary Information. In the bottom panels we show two sample countries from other regions of the World. In both cases, currently the epidemic is in the strolling regime after the first wave, indicating an imminent restart of the epidemic. In cases with an ongoing strolling, $S_t$ is fitted to reproduce the data.

Europe: Most of the European countries are being hit by a second wave of the COVID-19. A general trend we observe is that the infection rate during the second wave is smaller than the one of the first wave, as shown by the values of $\gamma _\text{eRG}$ in Supplementary Table T1 in the Supplementary Information. The comparison is done by fitting the two waves independently by use of the eRG model. Moreover, the expected cumulative number of infected cases during the second wave is much larger than that for the first wave, even taking into account the higher number of tests performed during the second wave. The emergence of the second wave was explained as arising from the interactions between countries^13^, nevertheless the presence of strolling between the two waves indicates that both effects participated. The decrease of the geographical uniformity indicator, shown in Fig. 1d, indicates that the strolling had an important role in diffusing the virus across the countries (for England, the uniformity was present since the first wave). The geographical diffusion may also be the reason behind the fact that the second wave has infected a larger portion of the population.

The CeRG model cannot be applied straightforwardly to the first two waves, due to the uncertainty in their relative normalisation, while it can be used to estimate when a third wave will hit starting from the data of the current (second) wave. The result is shown in Table 1, and in Fig. 3 and the top row of Fig. 4 (additional plots are provided in the Supplementary Information). The timing of the next wave peak depends crucially on the time-dilation parameter $\gamma$, and on the amount of strolling in the intermediate endemic phase: in the projections, we fitted $\gamma$ to reproduce the second wave and fixed $S_\text{t} = 0.01$, with the corresponding total number of new cases, expected during the strolling, reported in the figures. The last column of Table 1 shows that a wide range of peak timings are expected, ranging from March to November 2021, where we associated an error of 1 week to the projection due to a variation of 10% in the infection rate. These results show clearly that controlling the infection rates and reducing the level of strolling after the end of the second wave are keys to delaying the next wave. Another element that should be included is the number of travellers across various countries^13^, which can help propagate the wave from country to country, thus affecting the ones with pronounced delayed projections.

In the right panel of Fig. 2 we show the delay between the current and next waves (in weeks) for 5 European countries, as a function of the daily number of new cases during the strolling. Thus, to delay the next wave peak beyond 40 weeks from the current one, it would suffice to keep the number of new cases below 10 per day. Delays beyond 20 weeks can be obtained with 100 new cases per day per million inhabitants. France has comparatively larger delays due to the smaller $\gamma$ obtained from the data.

The US: The US has already seen two waves in April and July–August, and is undergoing a third. However, the first two waves are geographically distinct, with the episode in April mainly involving New York and New England, and the second spreading all over the remaining states. This is well illustrated by the geographical uniformity indicator in Fig. 1d, which sharply drops between the two episodes. The third point, based on the data of November, is still preliminary and will decrease as the third wave approaches its peak. To analyse the evolution of the COVID-19 epidemic in the US, a dedicated study which takes into account sub-regions is required. The uniformity analysis suggests that the first two episodes should be described in terms of interactions between states, while the third one may be originated by the strolling. Results of this analysis and projections for the future waves in the US will be presented in a separated article^22^.

Other countries: We included in our analysis a selection of countries for other regions of the World, selected in order to represent all continents. Note that we retained only countries for which the multiwave analysis is best explained in terms of the CeRG model, i.e. where diffusion of the virus in sub-regions do not produce features that would require a multi-region analysis. The latter situation can be tackled within coupled CeRG equations, but this analysis goes beyond the scope of the present work.

In Table 1 we show the results for the selected countries, also illustrated in the bottom row of Fig. 4 for South Africa and Saudi Arabia. In most selected cases, the country is in the strolling regime, following the end of the first or second wave, thus allowing us to tune the CeRG parameters to reproduce the strolling and give a more reliable forecast for the following wave. In some cases, like South Africa, the high level of strolling indicates that a new wave is imminent. As we are not trying to perform a fit, due to the many uncertainties in the social distancing and testing policies, the scenario we present should be considered as a probable one. Yet, it should be noted that the model does not leave much room to modify the expected number of total infected cases during the future wave nor change the timing substantially, without drastic pharmaceutical or non-pharmaceutical interventions.

## Discussion

We provide a mathematical understanding of the wave pattern for pandemics, like the COVID-19 one. The approach is employed to forecast the timing of a future wave based on the number of new infections during the intermediate endemic phase. The timing of the new wave is related to a newly introduced parameter, $S_t$, that can be easily deduced from the cumulative number of infected cases. We studied several countries in different regions of the World and, in absence of any pharmaceutical interventions, we estimate the timing of the next wave of infections. We found countries where a new wave will start in December 2020, like South Africa, and countries where it could start as late as October 2021, like in France. Our predictions will be affected by the border control regulations with the generic effect of inducing an early increase in the number of infections.

Our understanding of the wave structure of the COVID-19 pandemic draws the attention to the inter-wave *strolling* period. We discover that controlling the number of new infections during the strolling period is necessary to delay the beginning of a future wave. This amounts to imposing social distancing measures and break potential chains of infections after the end of the wave in order to keep $S_t$ as low as possible. Delaying the next wave is crucial in order to have enough time to realise an effective vaccine campaign.

Our results can effectively guide policymakers to time (non)pharmaceutical interventions to delay or reduce the impact of future COVID-19 waves. Until now, most measures are taken when the number of new infected cases has already grown substantially. At this point in time one can only contain the wave, not avoid it, with serious impact for the loss of human lives as well as the economy. We prove that intervening during the strolling period of endemic diffusion is essential to delay or avoid a new wave while buying time for pharmaceutical interventions, like an effective vaccine campaign. More specifically, to maximise the delay, the strolling parameter must be kept small, $S_t \approx 10^{-5}$ for an optimal use of the enacted measures. In most countries, this implies that the number of new cases at the end of the wave should be kept at the level of 10 cases per million inhabitants per day. This effect can be achieved by keeping or introducing new measures after the end of the wave, in the form that is more appropriate for the local conditions.

## Supplementary Information

Supplementary Information.
