# Supplementary material for: Multiwave pandemic dynamics explained: how to tame the next wave of infectious diseases
Source: Sci Rep. 2021 Mar 23;11:6638. doi: 10.1038/s41598-021-85875-2 (PMC7988059; doi:10.1038/s41598-021-85875-2)
Supplement: Supplementary file 1 — Supplementary Information. [file 41598_2021_85875_MOESM1_ESM.pdf]

Multiwave pandemic dynamics explained: How to tame the next wave of infectious diseases

Giacomo Cacciapaglia[1,2,*,+], **Corentin Cot**[1,2,+], **and Francesco Sannino**[3,4,*,+]

[1]Institut de Physique des 2 Infinis (IP2I), CNRS/IN2P3, UMR5822, 69622 Villeurbanne, France
[2]Université de Lyon, Université Claude Bernard Lyon 1, 69001 Lyon, France
[3]CP3-Origins & the Danish Institute for Advanced Study, University of Southern Denmark, Campusvej 55, DK-5230 Odense, Denmark
[4]Dipartimento di Fisica E. Pancini, Università di Napoli Federico II & INFN sezione di Napoli, Complesso Universitario di Monte S. Angelo Edificio 6, via Cintia, 80126 Napoli, Italy
[*]g.cacciapaglia@ipnl.in2p3.fr, sannino@cp3.sdu.dk
[+]these authors contributed equally to this work

ABSTRACT

Pandemics, like the 1918 Spanish Influenza and COVID-19, spread through regions of the World in subsequent waves. Here we propose a consistent picture of the wave pattern based on the epidemic Renormalisation Group (eRG) framework, which is guided by the global symmetries of the system under time rescaling. We show that the rate of spreading of the disease can be interpreted as a time-dilation symmetry, while the final stage of an epidemic episode corresponds to reaching a time scale-invariant state. We find that the endemic period between two waves is a sign of instability in the system, associated to near-breaking of the time scale-invariance. This phenomenon can be described in terms of an eRG model featuring complex fixed points. Our results demonstrate that the key to control the arrival of the next wave of a pandemic is in the strolling period in between waves, i.e. when the number of infections grow linearly. Thus, limiting the virus diffusion in this period is the most effective way to prevent or delay the arrival of the next wave. In this work we establish a new guiding principle for the formulation of mid-term governmental strategies to curb pandemics and avoid recurrent waves of infections, deleterious in terms of human life loss and economic damage.

Methods

CeRG approach to multi-wave dynamics

In the original *eRG* approach[1], rather than the number of cases, it was used its natural logarithm $\alpha(t) = \ln I(t)$. For a single wave pandemic this provides a good fit to the data. To describe multiple waves[2], it is better to use the eRG directly for the cumulative number of total cases rather than its log. The derivative of $I(t)$ with respect to time is interpreted as the *beta function* of an underlying microscopic model. In statistical and high energy physics, the latter governs the time (inverse energy) dependence of the interaction strength among fundamental particles. Here it regulates infectious interactions.

Thus, the dictionary between the eRG equation for the epidemic strength $I(t)$ in an isolated region of the world[1] and the high-energy physics analog is

$$-\beta_{\rm eRG}(I(t)) = \frac{dI(t)}{dt} = \gamma I \left(1 - \frac{I}{A}\right)^{2p}, \qquad \text{(E1)}$$

whose solution, for $2p = 1$, is a familiar logistic-like function

$$\frac{I(t)}{A} = \frac{e^{\gamma t}}{b + e^{\gamma t}}. \qquad \text{(E2)}$$

This solution can effectively describe one wave of the epidemic, and is related to the SIR model via time-dependent parameters[3]. The dynamics encoded in Eq. (E1) is that of a system that flows from an Ultra-Violet fixed point at $t = -\infty$, where $I = 0$, to an Infra-Red one where $I = A$, with A being the total number of infected cases at the end of the wave. The parameter γ is an effective infection rate, characterising the diffusion slope. Both the equation and the solution show that γ can be absorbed in a redefinition of time, $\tau = \gamma t$, so that γ can also be interpreted as a *time-dilation* factor, which differs by region and depends on external factors (social, demographic and relative to non-pharmaceutical interventions) which can slow down or accelerate the diffusion of the virus. Finally, b shifts the solution in time to match the beginning of the epidemic in each region, as shown by

the equivalence $b \to 1$ with $\gamma t \to \gamma t - \ln b$. Further details, including what parameter influences the *flattening of the curve* and location of the inflection point and its properties can be found in[4] and in[1].

The presence of a truly interacting fixed point at large times predicts that the total number of cases approaches a constant value, while the new cases drop to zero. This feature can be associated to the system approaching a time scale-invariant state. Most regions affected by COVID-19, however, show a slightly different dynamics: in fact, the infection stabilises to an endemic period of constant growth, where the total cases continue growing linearly. This region can be associated to a breaking of the time scale-invariance[2], which does not allow the system to approach the fixed point and generates the *strolling* regime of linear growth. To account for this feature, the beta function in (E1) can be extended as follows:

$$-\beta_{\text{CeRG}}(\iota) = \frac{d\iota}{d\tau} = \iota \left[(1-\iota)^2 - \delta\right]^p , \tag{E3}$$

where we have used normalised variable $\iota = I/A$ and $\tau = \gamma t$. The two new parameters δ and p are real numbers, with p positive. For negative δ, the second factor has complex zeros, so that the system cannot approach an IR fixed point but, for small $|\delta|$, it lingers near the would-be fixed point $\iota \approx 1$ for a long time, inversely proportional to $|\delta|$. This feature can explain the origin of the strolling[2]. In this work, we will use the new Complex eRG (CeRG) approach to describe multi-wave dynamics.

The solution of Eq. (E3) still encodes a single wave, followed by a strolling period. The latter signals an instability of the system, and precedes a new exponential increase of new cases. The mathematical model can be extended to describe multiple waves by endowing the CeRG beta function with new (quasi) zeros as follows:

$$-\beta_{\text{multiwaves}}(\iota) = \iota \left[(1-\iota)^2 - \delta_0\right]^{p_0} \prod_{\rho=1}^{w} \left[(1-\zeta_\rho \iota)^2 - \delta_\rho\right]^{p_\rho} , \tag{E4}$$

with $\zeta_\rho < 1$, $0 < -\delta_\rho \ll 1$ and $p_\rho > 0$. We have that $w+1$ is the total number of pandemic waves, and that the diffusion will stop if $\delta_w = 0$, with a total number of infected cases equals $I(\infty) = A/\zeta_w$.

For a case with two waves, $w = 1$, the value of ι at the two peaks (where the new infected cases reach a local maximum) can be determined by the zeros of the derivative of the beta function, $\partial\beta_{\text{multiwaves}}/d\iota = 0$. Setting $\delta_i = 0$ for simplicity (and because their value is very small and numerically irrelevant), we find

$$\iota_{\max}^{0/1} = \frac{1+\zeta_1+2p_0+2p_1\zeta_1}{2\zeta_1(1+2p_0+2p_1)} \left(1 \mp \sqrt{1-2\frac{2\zeta_1(1+2p_0+2p_1)}{(1+\zeta_1+2p_0+2p_1\zeta_1)^2}}\right) . \tag{E5}$$

From the definition of the beta-function, the time between the two peaks can be computed as follows:

$$\Delta\tau_{\text{peaks}} = \int_{\iota_{\max}^0}^{\iota_{\max}^1} \frac{dx}{-\beta_{\text{multiwaves}}(x)} . \tag{E6}$$

Note that the dependence on δ_1 is negligible, because the domain of the integral is always far from the second set of complex zeros. The value of δ is directly related to the number of new cases during the strolling, given by

$$S_{\text{t}} = \left.\frac{d\iota}{d\tau}\right|_{\text{strolling}} \approx -\beta_{\text{multiwaves}}(1) = (-\delta_0)^{p_0}(1-\zeta_1)^{2p_1} . \tag{E7}$$

Interacting regions

Interactions among different regions of the world can be taken into account by adding to the beta function for each country j the following interaction term[5]:

$$\frac{\delta I_j(t)}{A_j\, \gamma_j \delta t} = \sum_l \frac{k_{jl}}{\gamma_j\, n_{mj}} \frac{I_l(t) - I_j(t)}{A_j} , \tag{E8}$$

where n_{mj} is the population of region-j in millions. The matrix k_{jl} is proportional to the numbers of travellers between each of the regions considered in the interaction.

Extracting the parameters from the data

We first fit the first and second wave (if reached) by use of independent solutions to the eRG framework, with numerical results shown in Table T1. This exercise will allow us to compare the two waves, in particular the infection rates γ_{eRG}, which are independent on the normalisation of the cumulative number of infected. We use open-source data from the online repository

	eRG fits				
country	1st wave		strolling	2nd wave	
	A	γ_{eRG}	I'_{strol}	A	γ_{eRG}
France	2034(13)	0.135(3)	5.2(8)	$61(6) \cdot 10^3$	0.048(5)
Italy	3638(28)	0.100(3)	3.5(4)	$41(2) \cdot 10^3$	0.075(8)
UK	3943(25)	0.0794(15)	9.1(2)	$21.3(2) \cdot 10^3$	0.0717(8)
Germany	2006(14)	0.128(3)	5.1(2)	$20(3) \cdot 10^3$	0.058(2)
Spain	5000(30)	0.134(4)	7(1)	$30(4) \cdot 10^3$	0.067(1)
Switzerland	3304(22)	0.160(4)	10(2)	$40(4) \cdot 10^3$	0.10(1)
Netherlands	2546(15)	0.112(2)	10.3(2)	$26.1(2) \cdot 10^3$	0.0797(8)
Belgium	4758(27)	0.116(2)	22.1(6)	$40.7(4) \cdot 10^3$	0.121(2)
Denmark	1950(10)	0.088(2)	6.7(1)	$12(2) \cdot 10^3$	0.062(2)
Iceland	5285(16)	0.176(2)	3.6(2)	$9.9(1) \cdot 10^3$	0.087(2)
Canada	2643(20)	0.0732(14)	8.2(2)	$16(2) \cdot 10^3$	0.046(5)
South Africa	11039(35)	0.0704(5)	26.69(9)	–	–
Bolivia	12240(20)	0.0442(2)	9.2(3)	–	–
Saudi Arabia	9560(20)	0.0447(3)	11.51(3)	–	–
Australia	255(1)	0.239(5)	0.38(2)	735(3)	0.1015(7)
Japan	130.7(7)	0.125(2)	0.22(2)	434(5)	0.094(1)
South Korea	185(2)	0.225(14)	0.569(8)	157(3)	0.136(6)

Table T1. Numerical results for the eRG fits. Fit of the first two waves using the eRG model[1], where A are indicated in number of cases per million inhabitants, γ_{eRG} is given in inverse days, and the new parameter I'_{strol} indicates the number of daily new cases per million inhabitants obtained by fitting the strolling period with a linear growth. The values highlight that the second wave typically features a smaller infection rate and a larger number of infected cases.

Ourworldindata.org. We first identify the first wave, within the dates indicated in Table T2, and fit the data by use of a simple logistic function:

$$I(t) = \frac{A e^{\gamma_{eRG} t}}{b + e^{\gamma_{eRG} t}} \,. \tag{E9}$$

We then fit the data after the end date of the first wave (having subtracted the final number of cases) with a second independent logistic function plus a linearly growing term, which characterises the strolling period. If the second wave is absent, we simply fit the linear strolling. It is well known[1] that the fit is optimal only after the peak is achieved, so we tune the fit to reproduce the expected peak in some cases. In two cases we take into account spurious features in the data: for France, a sudden drop in the number of new cases can be observed after November 8, which is accountable by a decrease in the number of tests plus a reduced positivity rate; for Spain, the initial growth is due to a hotspot in Catalunya, followed by the spread to the rest of the country. Conservatively, we only fit the second higher sub-peak.

As a second step, we tune the CeRG model, without interactions, to reproduce the last wave in order to forecast the arrival of the next one. The beginning of the wave (after subtracting any initial data) allows to determine γ, while A_0 and p_0 are determined by the end of the wave and the peak region. For countries where the second wave is still ongoing, we fix $p_0 = 0.55$, while its value is fitted in other cases. Furthermore, we fix the strolling parameter $S_t = 0.01$, or fit it to the data for countries that are currently in the strolling regime. The parameters obtained by this tuning are reported in Table T2, and have been used to obtain the peak dates and in the plots.

Geographical uniformity indicator

To investigate potential differences between waves due to the geographical spread of the virus, we define an uniformity indicator for each country by studying the number of new cases in various regions over a period of time around the peak. The indicator is inspired to a χ^2-variable, measuring how far is the actual distribution of cases compared to a perfectly uniform distribution, and it is defined as follows :

$$\chi^2(\Delta t) = \frac{1}{n_r} \sum_{i=1}^{n_r} \left(\frac{I'_{ri}(\Delta t)}{\langle I'_r(\Delta t) \rangle} - 1 \right)^2 , \tag{E10}$$

	CeRG parameters							
country	A	γ	δ_1	p_0	p_1	ζ_1	1st wave start	1st wave end
France	$61 \cdot 10^3$	0.058	$1.0 \cdot 10^{-3}$	0.55	0.6	0.5	2020-02-28	2020-05-08
Italy	$41 \cdot 10^3$	0.091	$1.0 \cdot 10^{-3}$	0.55	0.6	0.5	2020-02-18	2020-05-18
UK	$26 \cdot 10^3$	0.082	$1.0 \cdot 10^{-3}$	0.55	0.6	0.5	2020-03-09	2020-06-17
Germany	$22 \cdot 10^3$	0.070	$1.0 \cdot 10^{-3}$	0.55	0.6	0.5	2020-03-09	2020-05-18
Spain	$36 \cdot 10^3$	0.078	$1.0 \cdot 10^{-3}$	0.55	0.6	0.5	2020-02-28	2020-05-18
Switzerland	$40 \cdot 10^3$	0.127	$1.0 \cdot 10^{-3}$	0.55	0.6	0.5	2020-03-09	2020-04-28
Netherlands	$31 \cdot 10^3$	0.092	$1.0 \cdot 10^{-3}$	0.55	0.6	0.5	2020-03-09	2020-05-18
Belgium	$45 \cdot 10^3$	0.157	$1.0 \cdot 10^{-3}$	0.55	0.6	0.5	2020-03-09	2020-05-18
Denmark	$15 \cdot 10^3$	0.071	$1.0 \cdot 10^{-3}$	0.55	0.6	0.5	2020-03-04	2020-05-28
Iceland	$11 \cdot 10^3$	0.114	$1.0 \cdot 10^{-3}$	0.55	0.6	0.5	2020-03-04	2020-04-28
Canada	$17 \cdot 10^3$	0.055	$1.0 \cdot 10^{-3}$	0.55	0.6	0.5	2020-03-19	2020-06-17
South Africa	$12 \cdot 10^3$	0.102	$2.2 \cdot 10^{-3}$	0.56	0.6	0.45	2020-04-16	2020-09-03
Bolivia	$13 \cdot 10^3$	0.057	$1.2 \cdot 10^{-4}$	0.51	0.6	0.45	2020-04-20	2020-10-27
Saudi Arabia	$10 \cdot 10^3$	0.058	$1.3 \cdot 10^{-3}$	0.51	0.6	0.5	2020-03-21	2020-10-07
Australia	772	0.122	$8.4 \cdot 10^{-6}$	0.52	0.6	0.5	2020-02-28	2020-04-23
Japan	500	0.103	$4.9 \cdot 10^{-2}$	0.7	0.6	0.4	2020-02-28	2020-05-18
South Korea	180	0.191	$5.7 \cdot 10^{-3}$	0.6	0.6	0.45	2020-02-18	2020-04-08

Table T2. Parameters for the CeRG forecast for the next waves. Fit of the current wave using the CeRG model[2], with parameters expressed in the same units as in Table T1. For all countries, the current wave corresponds to the second, except for South Africa, Bolivia and Saudi Arabia for which the first one is considered. The dates in the last two columns indicate the period where the first wave data was not considered in the fits (except for South Africa, Bolivia and Saudi Arabia).

where $I'_{ri}(\Delta t)$ is the number of new infected cases in the region ri over a time interval Δt, and $\langle I'_r(\Delta t)\rangle$ indicates the mean over the whole set of regions. We normalise the parameter to the number of regions in order to obtain comparable values. The smaller is χ^2, the more uniform is the diffusion of the virus. We also noted that the value of the uniformity indicator reaches local minima at the peak of each wave, thus the values reported for starting waves are larger than the final one.

To compute the values, we only used open-source data available on various online repositories. For Japan, we obtained the local data for the 46 prefectures from Toyokeizai.net. In the analysis we omitted Okinawa because of its distance from the mainland. For Germany (16 Landen), Italy (20 regions), Spain (17 regions) and England (9 regions), we obtained data collected in periods of about 4 weeks from Citypopulation.de. For France, as the local data on the new number of infected is not available before May 13, 2020, we used information for the number of hospitalisations in the 94 continental departments from Data.gouv.fr. Finally, the data for each state of the US is obtained from Ourworldindata.org.

For Japan and the US, the result for the third wave overestimates the one at the peak, which is not reached yet.

Additional plots

In Figs S1 and S2 we provide a visualisation of the CeRG forecast for the next wave for the countries not shown in the main text.

References

1. Della Morte, M., Orlando, D. & Sannino, F. Renormalization Group Approach to Pandemics: The COVID-19 Case. *Front. Phys.* **8**, 144, DOI: https://doi.org/10.3389/fphy.2020.00144 (2020).

2. Cacciapaglia, G. & Sannino, F. Evidence for complex fixed points in pandemic data, DOI: https://doi.org/10.21203/rs.3.rs-70238/v1 (2020). 2009.08861.

3. Della Morte, M. & Sannino, F. Renormalisation Group approach to pandemics as a time-dependent SIR model (2020). 2007.11296.

4. McGuigan, M. Pandemic modeling and the renormalization group equations: Effect of contact matrices, fixed points and nonspecific vaccine waning (2020). 2008.02149.

5. Cacciapaglia, G. & Sannino, F. Interplay of social distancing and border restrictions for pandemics (COVID-19) via the epidemic Renormalisation Group framework. *Sci Rep* **10**, 15828, DOI: https://doi.org/10.1038/s41598-020-72175-4 (2020). 2005.04956.

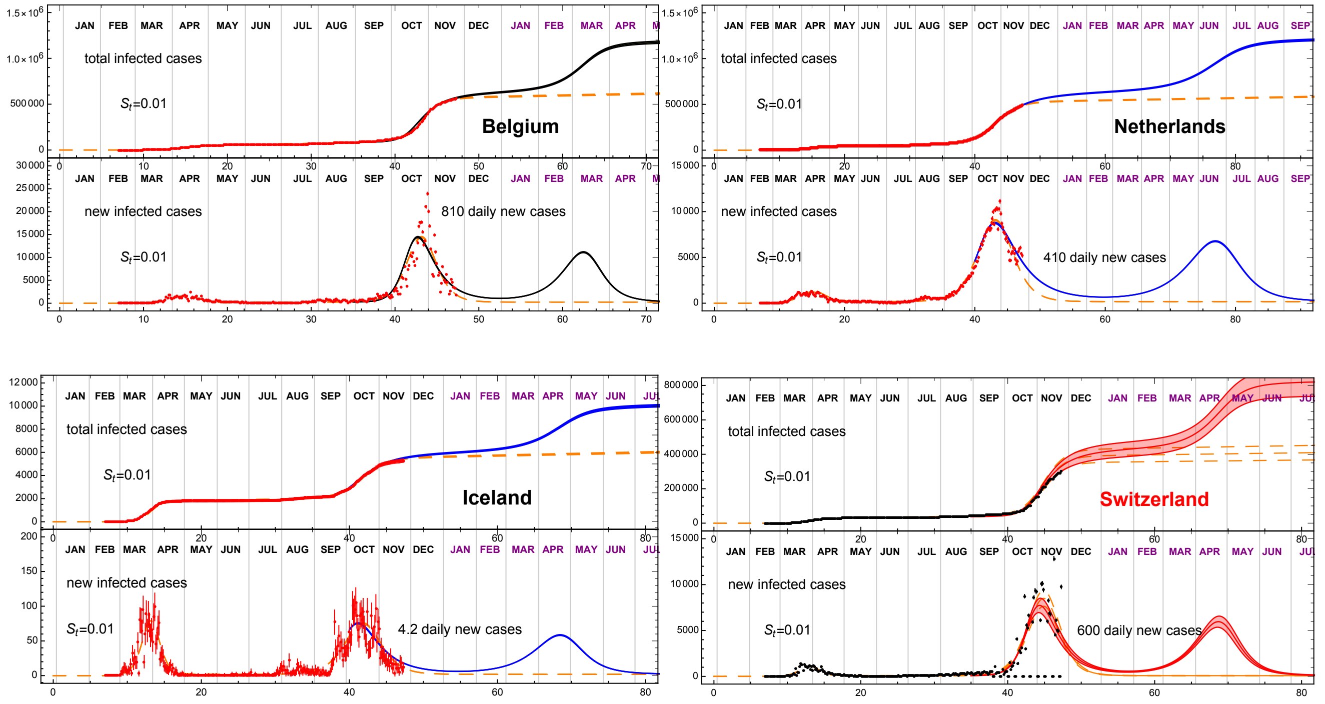

Figure S1. Strolling as a precursor of a COVID-19 third wave. Plots showing the CeRG model forecast for the future wave for a selection of European countries. The prediction corresponds to $S_{\mathrm{t}} = 0.01$, and is compared to the data (adjourned to November 23) and the eRG fit from Table T1 (dashed).

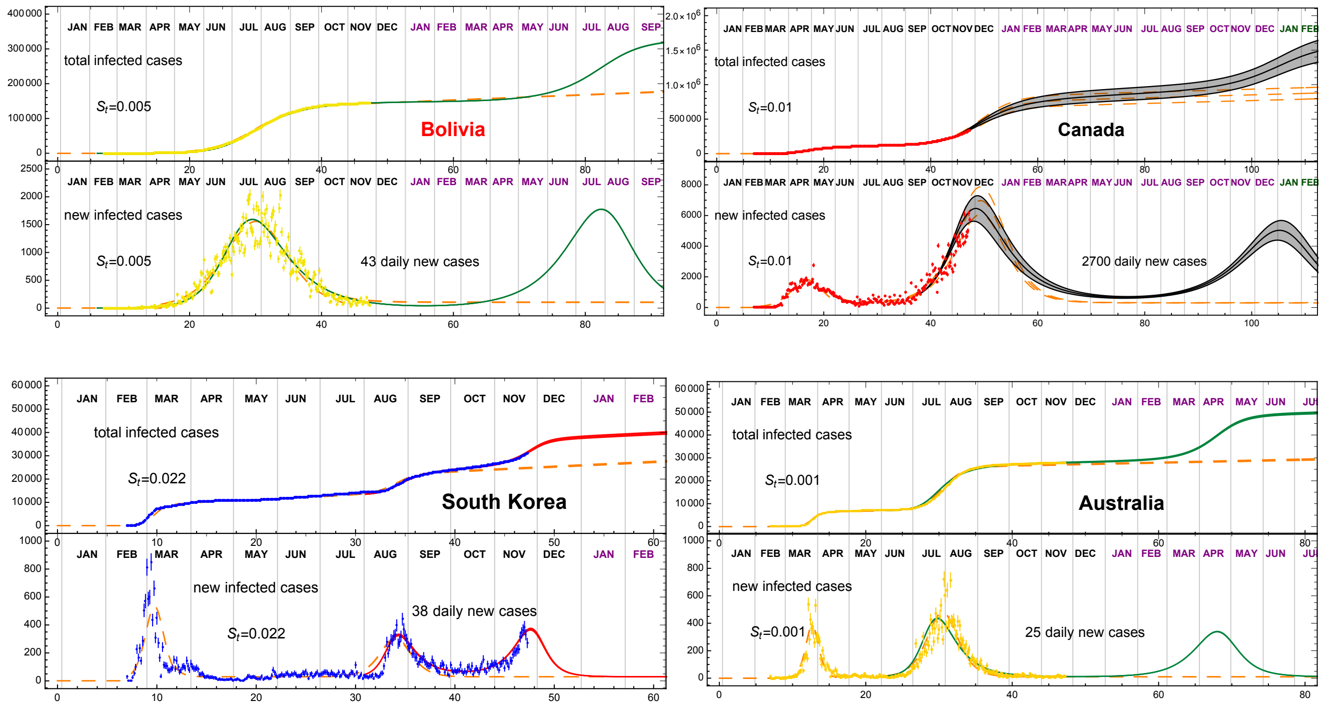

Figure S2. Strolling as a precursor of a COVID-19 third wave. Same as Fig. S1 for the remaining non-European countries. The prediction corresponds to $S_t = 0.01$ for Canada, while the strolling parameter is fitted to the data for Bolivia, South Korea and Australia, and is compared to the data (adjourned to November 23) and the eRG fit from Table T1 (dashed).
